# The International Landscape of Medical Licensing Examinations: A Typology Derived From a Systematic Review

**DOI:** 10.15171/ijhpm.2018.32

**Published:** 2018-04-28

**Authors:** Tristan Price, Nick Lynn, Lee Coombes, Martin Roberts, Tom Gale, Sam Regan de Bere, Julian Archer

**Affiliations:** ^1^Collaboration for the Advancement of Medical Education Research & Assessment (CAMERA), University of Plymouth, Plymouth, UK.; ^2^Peninsula Schools of Medicine & Dentistry, University of Plymouth, Plymouth, UK.; ^3^School of Medicine, Cardiff University, Wales, UK.

**Keywords:** Assessment, National Licensing Examinations, Regulation, Workforce Planning

## Abstract

**Background:** National licensing examinations (NLEs) are large-scale examinations usually taken by medical doctors close to the point of graduation from medical school. Where NLEs are used, success is usually required to obtain a license for full practice. Approaches to national licensing, and the evidence that supports their use, varies significantly across the globe. This paper aims to develop a typology of NLEs, based on candidacy, to explore the implications of different examination types for workforce planning.

**Methods:** A systematic review of the published literature and medical licensing body websites, an electronic survey of all medical licensing bodies in highly developed nations, and a survey of medical regulators.

**Results:** The evidence gleaned through this systematic review highlights four approaches to NLEs: where graduating medical students wishing to practice in their national jurisdiction must pass a national licensing exam before they are granted a license to practice; where all prospective doctors, whether from the national jurisdiction or international medical graduates, are required to pass a national licensing exam in order to practice within that jurisdiction; where international medical graduates are required to pass a licensing exam if their qualifications are not acknowledged to be comparable with those students from the national jurisdiction; and where there are no NLEs in operation. This typology facilitates comparison across systems and highlights the implications of different licensing systems for workforce planning.

**Conclusion:** The issue of national licensing cannot be viewed in isolation from workforce planning; future research on the efficacy of national licensing systems to drive up standards should be integrated with research on the implications of such systems for the mobility of doctors to cross borders.

## Introduction

The regulation and licensing of doctors within any particular country is the result of economic, political, geographic and demographic factors.^1^ Highly developed countries have witnessed an identifiable trend towards an increase in medical regulation over the last 20 years. In large part this has been in response to the increasing demands of accountability and assurance of public trust, and as regulation has increased so has the demand for large scale examinations and assessments as a means of assuring minimum standards.^2^ Much of the debate, including recent contributions to Academic Medicine,^3-5^ has focused on the efficacy of national licensing and whether current examination systems are appropriately preparing physicians for independent practice. This paper reports a typology, arising from a systematic review, which broadens this debate by considering the wider context which has shaped the policies and debates concerning national licensing, namely: The internationalization of medical training, increased medical workforce mobility and, relatedly, workforce planning.

The internationalization of medical training refers to the recent proliferation of medical schools and the mobility of medical students. Globally, it has been reported that between 1995 and 2003 the number of medical schools has increased by around 54%^6^ and has been rising ever since.^7^ In some respects this is part and parcel of the impact of globalization on higher education more generally, driven by both the free market giving students the opportunity to choose to study abroad, and the managed market whereby government policies have been aimed at procuring higher education for its citizens from abroad in order to meet workforce needs.^8^

Relatedly, there has also been a marked increase in the mobility of the medical workforce, aided on the supply side by the internationalization of medical training, with doctors and other healthcare professionals increasingly familiar with the systems of countries other than those in which they trained. In addition, free trade agreements, most notably in the European Economic Area (EEA: The European Union plus some of the non-EU European states party to the European Free Trade Association – Norway, Lichtenstein, Switzerland, and Iceland), have sought to create internal markets predicated on the free movement of labor.^9^ On the demand side, in highly developed economies there is an almost universal shortage of qualified doctors,^10^ with medical schools under strain to cope with the rising demand for places. The net effect of this has been an increasing number of doctors who have trained under a system with different curricula, regulatory and cultural norms than the one in which they end up practicing. Concerns have repeatedly been raised about marked differences in the standards in medical education internationally^11^ and in some cases that students are being admitted to medical schools overseas who would not have gained entry in their domiciled nation.^12^ As a result medical leaders are having to view standardization and regulation through new “global glasses.”^9^

In addition to the dynamics of globalization, neo-liberal systems of management, where the state seeks to promote its control of professionals from a distance, has resulted in the centralization and standardization of assessment methodologies.^2,13,14^ One major catalyst in this process has been high profile scandals that have increased public pressure for greater regulation of the health professions.^15^ One answer to the question of how to standardize medical regulation in a globalized environment is through large scale national licensing exams, taken by students on or shortly after graduation, and depending on the system in question, required of doctors who have trained overseas.

The subsequent drive towards standardization has brought into focus the evidence base for national and other large scale licensing examinations. Opinion on this subject is divided,^16,17^ with the arguments supported by a small evidence base.^18-20^ Some academics and educationalists argue that all new doctors should be tested to make sure that they have achieved a minimum standard,^21^ and that all must pass to enter the profession so it is ‘fair.’^19^ A further argument for pooling resources and expertise is that it drives up testing standards and is cost effective.^22,23^

These arguments for better collaborative working have been driven in part by concerns that medical school assessment is unstandardized,^24^ and that this makes an unfair difference to subsequent performance with testing,^25,26^ and more importantly in practice.^27-30^

However national licensing examinations (NLEs) are nothing new with the United States Medical Licensing Examination (USMLE), and Medical Council of Canada Qualifying Examination (MCCQE) world leading examples.^31-33^ Although some recent studies have shown correlations between NLE scores and both patient outcomes^31^ and complaints,^28^ this does not in itself support the assertion that NLEs lead to better doctors.^34,35^ One of the confounding variables identified is the evidence suggesting that those who get the highest scores are likely to get the best jobs, and it is this working environment that may lead to both better patient outcomes and fewer complaints.^36-38^ A recent systematic review of national licensing found that the existing evidence base points to a correlation between NLE exam scores and better performance in later career, but that there is no solid evidence that adopting a national licensing exam will drive up standards.^34^

At the same time there are concerns that standardizing medical education in this way will reduce innovation and advancements in curricula,^18,39,40^ and that NLEs are now out-of-date with the new modalities of testing throughout medical school and more recently in practice.^4,5,16^ Some commentators have suggested that learning outcomes that are easily testable simply become the focus of NLEs, and that those competences do not correlate well with actual practice.^17,18,41^ Others have focused on the consequences of NLEs for candidates, including stress and burnout as students compete for the highest grades.^27^

In the United Kingdom, the General Medical Council (GMC) – the medical regulator – announced that they were planning to develop and implement a new Medical Licensing Assessment (MLA) by 2021.^42^ As part of the process they commissioned a review of the international literature,^43^ with one of the objectives of the review to understand what currently happens in large scale testing of doctors. While the issue of quality in relation to national licensing is a central issue, this has been covered elsewhere.^1,34,35^ This paper contributes to the debates over NLE’s by addressing the following research question: what different types of national licensing system are used in countries comparable to the United Kingdom and what are the implications of these systems for workforce planning within these countries?

To address this question, this paper develops a typology for national medical licensing based on candidacy, ie, who undertakes the exam that can be used for comparison across systems.

## Methods

Information on NLEs is substantial but dispersed. As such, to answer the central research question, three different sources of information were searched. Firstly, and in line with the broader aims of the research commissioned by the GMC, a systematic review of the national medical licensing literatures was conducted.^34^ The following databases were searched: BMJ, Embase, Medline, PubMed, PsychINFO, Science Direct, and Wiley Online. These were chosen based on the prior knowledge and experience of the research team. The specific search terms included were ‘national licensing examinations for doctors,’ ‘national licensing exams for doctors,’ ‘international medical graduate examinations,’ ‘international medical graduates,’ ‘IMGs,’ ‘international medical graduate programmes,’ ‘accreditation,’ ‘credentialing,’ ‘registration,’ and ‘certification.’

A wide range of literature was sought in order to capture as much data as possible. For this reason, there were no restrictions on language or quality. To ensure that our findings were based on the most up-to-date literature, and because NLE debates have evolved rapidly in recent years, only literature since 2005 was included in the review. The full inclusion and exclusion criteria is listed in Table 1.

**Table 1:** Inclusion and Exclusion Criteria^a^

**Inclusion Criteria**	**Exclusion Criteria**
Medicine and healthcare professionals National or regional (State level) Exams taken early in career/soon after graduation Success in examination linked to ability to practise Any language (assuming translation could be obtained) Countries comparable to the UK using the UNDP development index Published since 2005	Outside healthcare Local or institutional level Specialist examinations Prior to 2005

Abbreviation: UNDP, United Nations Development Programme. ^a^ Adapted from Archer et al.^43^

Data from included papers were extracted by the team using a standardized data extraction form (Supplementary file 1). Some of this information, such as the evidence for validity, related to the wider aims of the GMC commissioned study. For the purposes of this study, the important information extracted related to the population, ie, who takes the exams, and the characteristics of the examinations.

In order to further develop the typology of NLEs that is presented in this paper, medical regulators’ websites or those bodies responsible for licensing doctors were reviewed. The search was limited to the 49 countries described by the United Nations (UN) as ‘very high human development.’^45^ This limitation was applied as it was assumed that only those countries with adequate resources would be likely to have a NLE. From each website any publicly available details were extracted about how doctors are licensed in that country and the process by which each regulator dealt with doctors who wished to live and work in their jurisdictions.

Finally, medical regulators in each country were contacted via letter, developed in collaboration with the GMC and the International Association for Medical Regulators and Authorities (IAMRA), in order to access any additional information such as assessment manuals or guidelines to give further insight into the types of system in operation. The final agreed letter and survey are included in Supplementary files 2 and 3.

### Analysis

The data from this paper are therefore derived from three sources: the academic literature, publicly available information from regulators’ websites, and a survey of medical regulators. All of these sources were used to inform the development of a typology in line with the central research question. Once the relevant information had been collated, in the analysis stage of research, the data were then sorted according to a candidacy typology. Typologies are widely used in healthcare research to compare and contrast different systems. They can take different forms, depending on how they are categorized and which factors are prioritized.^46,47^ For example, health systems are usually categorized according to economic determinants – ie, who pays^1^ – but alternative classifications based on political, cultural or organizational differences can be found.^1,48^

We developed the typology of NLEs to inform the debates around national licensing in a way that was relevant to the management of healthcare education and health workforce planning. As such, the decision was made from the outset to categorize the systems according to candidacy, ie, who takes the exam. However, the four types of candidacy we discuss below emerged inductively, from the process of engaging with the reviewed academic and grey literature.

## Results

The review of the literature contained 74 papers that either discussed the evidence for or implementation of NLEs (24 papers) or editorials, opinion pieces, and personal views written mostly by acknowledged experts in medical education and assessment (50 papers). The 24 papers with evidence for NLE’s (Supplementary file 4) were used to help populate the data for the typology of licensing examinations as well as to develop the more in-depth discussion on how these systems accommodate and are shaped by workforce planning considerations. Opinion pieces were used as background literature. A full breakdown of the search numbers of papers returned for each stage of the search process is provided in Figure.

**Figure:**
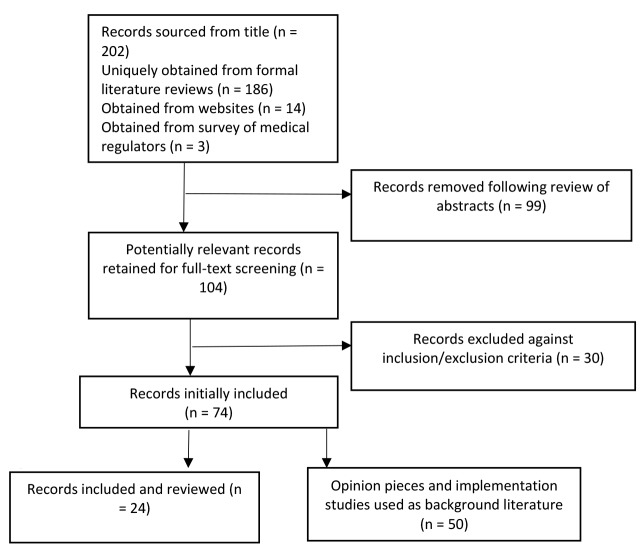
Overview of the Literature Search Process.

The website review in collaboration with the literature identified details for 23 of the 49 countries about licensing examinations. This was supported by 11 responses from regulatory authorities although none of the information provided went beyond what was already in the public domain. No regulatory authority highlighted an assessment manual or other offline material, despite the offer of anonymity, which provided an evidence base for their NLE, or any additional operational evidence.

The 23 countries whose online sites yielded details about a NLE are listed in Table 2. This includes information on the component parts of the NLE as well as the candidate type eg, international medical graduate only.

**Table 2:** NLEs and Component Parts^a^

**Country and Examination**	**Components**	**Candidacy**
Australia AMC	Part 1: AMC CAT. Tests knowledge of the principles and practice of medicine in general practice, internal medicine, pediatrics, surgery, obstetrics and gynecology. Candidates must pass this examination to go to take the AMC Clinical Examination. Part 2: AMC Clinical Examination: assesses clinical skills in medicine, surgery, obstetrics, gynecology, pediatrics, and psychiatry. Also assesses ability to communicate with patients, their families and other health workers.	***Type 3*** These are examinations on the Standard Pathway ie, the pathway for those IMGs who do not qualify for the other pathways into the Australian workforce.
Bahrain BMLE	Part 1: Written MCQ with a stem followed by 4 or five responses (1 correct answer). Followed by written MCQ test based on Patient Management Problems. Assesses clinical reasoning skill and ability. Part 2: OSCE followed by questions.	***Type 1*** Taken by all doctors who wish to practice in Bahrain.
Canada MCCQE	Part 1: Computer test consisting of MCQs followed by short menu, short answer questions on Clinical Decision-Making. Part 2: OSCE style simulation stations assessing competence, specifically knowledge skills and attitudes.	***Type 2*** IMGs and IMSs (along with home students) must pass the MCCEE.
Chile EUNACOM	Part 1: Written MCQs in different areas. Part 2: Practical examination of general practice. Clinical evaluation in a real or simulated environment in the areas of medicine, surgery, obstetrics- gynecology and pediatrics.	***Type 2*** Taken by all doctors who wish to practice in Chile. http://www.eunacom.cl/.
Croatia Croatian Medical Licensing Examination	No detail available.	***Type 1*** Taken by Croatian Graduates and non-EU/EEA nationals. EU/EEA nationals are exempt.
Finland: Professional Competence Examination	Part 1: Written exam on key areas of medicine. Part 2: Written exam on healthcare management. Part 3: Oral examination in a clinical setting (with patient present).	***Type 3***
France: Epreuves Classantes Nationales NCE (ranking examination)	Written theory test for national ranking.	***Type 1/2*** EU/EEA nationals can apply to take the ENC but non-EU/EEA IMGs must take a separate exam with limited places available.
Germany: *Staatsexamen*	Part 1: M1 *Physikum* or preclinical medicine after 2 years. Part 2: M2 written and oral practical includes the content of the entire clinical phase. MCQs.	***Type 1*** Only German doctors take these examinations. Non-EU/EEA nationals may be required to take a ‘knowledge test’ to prove their qualifications are equivalent to German standards.
Hong Kong: The Licensing Examination	Part 1: Examination in Professional Knowledge MCQs to test knowledge in basic science, medical ethics, community medicine, medicine, surgery, orthopedic surgery, psychiatry, pediatrics, obstetrics, and gynecology. Part 2: Proficiency Test in Medical English (scheduled for March 2015). Part 3: The Clinical Examination: to test how candidates apply professional knowledge to clinical problems. (scheduled for May/June 2015).	***Type 2*** All IMGs must pass Parts 1 and 2 to take Part 3.
Ireland: PRES	Part 1: All applicants undergo level 1 assessment and verification of their documentation. Those not exempt after this process go on to take the next parts. Part 2: Level 2: Computer-based examination using MCQs. Pass is required to move to level 3. Part 3: Assessment of Clinical Skills. OSCE style examination. Interpretation Skills test is one paper based examination.	***Type 3*** Non-EU/EEA graduates may be required to take a medical council examination unless exempt.
Israel	Written examination in Hebrew uses MCQs.	***Type 1/2*** For physicians who have graduated in Israel and from abroad. Requirement waived for physicians who have passed the USMLE. http://www.ima.org.il/ENG/Default.aspx.
Japan: NMLE	No detail available.	***Type 2*** Taken by all those who wish to work in Japan. Test is in Japanese. http://www.med.or.jp/english/.
Korea: KMLE	Part 1: Written examination. Part 2: OSCE-style clinical skills test.	***Type 2*** Overseas qualifications must be recognized by the Minister of Health & Welfare prior to IMGs taking the test.
New Zealand: NZREX (Clinical)	OSCs covering: history taking, clinical examination, investigating, management, clinical reasoning. Also, communication and professionalism is assessed.	***Type 3*** Eligibility requirements must be satisfied on each occasion. IMGs only.
Poland: State Physician and Dental Final Exam	The SP/DE is a written test, in Polish and consists of 200 MCQs only one correct answer out of the choices. Mix of medical knowledge, questions about specific medical processes, analysis of medical records, and establishing medical diagnosis.	***Type 1*** Content of the examination does not exceed the scope of the internship program. Oral skills are not tested. Medical schools test communication and procedural competencies.^49^ Taken by IMG and not EEA candidates.
Portugal: ‘Exame Nacional de Seriacao’ (Ranking examination for residency posts)	Written test MCQs on internal medicine.	***Type 1/3*** Medcial graduates sit a Portuguese licensing exam. IMGs must take a communication skills test.
Spain: MIR (National Residency Examination) ‘examen MIR’	Written test - MCQs	***Type 1*** Used for ranking medical students for specialty training.
Sweden: TULE-test	Part 1: Written test of medical knowledge. Part 2: Practical tests over 2 days.	***Type 3***
United Kingdom PLAB	Part 1: 200 single best answer questions Part 2: 18 scenarios OSCE	***Type 3*** For non-UK, EEA or Switzerland doctors medical school graduates who have no EC rights or an approved sponsor, or an approved postgraduate qualification eligibility to enter the GP or specialist register.
United States USMLE	Step 1: 322 MCQs to test and measure basic science knowledge. Consists of 7 blocks of 46 items. 1 hour for each block of test items. Maximum of 7 hours testing. Step 2: Clinical Knowledge test using MCQs and OSCE Clinical Skills test using standardized patients. Step 3: MCQs CCS followed by case simulations.	***Type 2*** IMGs must be certified by the ECFMGO to take USMLE Step 3 although individual jurisdictions may require extra training for IMGs of at least 1 year.
Qatar: Qualifying Examination	No detail available	***Type 1/2*** Graduates must pass the exam as must IMGs, but there is a waiver for certain recognised NLEs.
Switzerland: FLE	Part 1: Locally administered written exam using MCQs Part 2: OSCE style Clinical Skills examination.	***Type 1*** Swiss graduates must take the FLE. Non EU/EEA graduate qualifications are assessed at Cantonal level. IMGs take the test if they wish to practice independently.
UAE	No detail available	***Type 2*** Separate registration required to work in Dubai.

Abbreviations: AMC, Australian Medical Council; UAE, United Arab Emirates; IMSs, International medical students; MCCEE, Medical Council of Canada Evaluating Examination; EEA, European Economic Area; PRES, pre-registration examination system; USMLE, United States Medical Licensing Examination; NMLE, National Medical Licensing Examination; OSCE, objective structured clinical examination; CAT, computer adaptive test; ENC, Epreuves Classantes Nationales; MCQs, multiple choice questions; KMLE, Korean Medical Licensing Examination; PLAB, Professional and Linguistic Assessments Board; OSCs, Objective Structured Clinicals; FLE, Federal Licensing Examination; CCS, computer-based case simulation; ECFMGO, Educational Commission for Foreign Graduates; BMLE, Bahrain Medical Licensure Exam; MCCQE, Medical Council of Canada Qualifying Examination; IMG, international medical graduate. ^a^ Adapted from Archer et al.^43^

The literature on licensing examinations in medicine across the world is extensive, but not complete.^50-52^ However, the scope of the available literature was sufficient to capture some of the similarities and differences that exist within and between some of the 49 jurisdictions.

Categorized according to candidacy, essentially four different approaches to licensing examinations exist:

1. Where graduating medical students wishing to practice in their national jurisdiction must pass a national licensing exam before they are granted a license to practice;
2. Where *all* prospective doctors, whether from the national jurisdiction or international medical graduates (IMGs), are required to pass a national licensing exam in order to practice within that jurisdiction;
3. Where IMGs are required to pass a licensing exam if their qualifications are not acknowledged to be comparable with those of students from the national jurisdiction;
4. Where there is no NLE in operation.

The countries that have adopted the first approach are Germany,^53^ Switzerland, Poland,^54^ Bahrain,^55^ Qatar,^56^ and Croatia.^57^ All home trained students in these jurisdictions are required to pass the examination before they can apply for a license to practice. However, some IMGs may be exempted, eg, graduates from within the EEA are exempt from the licensing exams if they work within the EEA.

The second approach requires that any prospective doctor seeking to practice medicine within the national jurisdiction must pass the national licensing exam, regardless of where they have completed their medical training. This approach is used in a number of comparable countries, including Canada, Chile, Japan, Hong Kong, South Korea, the United Arab Emirates (UAE), and the United States. Most of the academic literature around these systems emanates from North America.^58^ It should be noted however that in some cases while the national licensing exam represents a minimum national standard, further training may be required of IMGs. For example, in the United States, where different jurisdictions set their own NLE pass rates, IMGs may have to undertake further training within that jurisdiction of at least a year before gaining a license.

The third category of licensing examination only targets IMGs and for this reason some may not perceive it be, strictly speaking, a “national” licensing exam. However, administration of these examinations is conducted at the national level, the examinations cover generic rather than specific skills, and success in the exam is necessary in order to gain a license to practice.

This approach is used in Australia^59^ and New Zealand,^60^ where comprehensive information is provided on the medical council websites, allowing prospective IMG doctors to determine what ‘pathway’ into the physician workforce their qualifications require them to follow. Both Australia and New Zealand, as well as the United Kingdom, currently operate an ‘accreditation’ model of licensing regulation. In each of these cases the prospective IMG doctors are required to evidence their language competence and to provide validated documentation of their primary medical qualifications. In the Australian system certain IMG qualifications are considered to have parity with those of graduates from Australia or New Zealand, whereas others are not. The process that establishes which qualifications are deemed acceptable is not always straightforward.

In Europe, those countries that are EEA member states are constrained by directives that stipulate the free movement of citizens across member states. This has implications for this third category of licensing examination as it is limited to only those who hold passports from outside the EEA, referred to here as non-EEA IMGs.^50^ For these non-EEA IMGs it means that across much of Europe they are required to undertake a range of examination processes to gain a license to practice in their chosen country. Some argue that these processes are flawed, partly because there is often little information available in many of the EEA member states concerning their licensing processes and there appears a lack of consistency in implementation and quality assurance.^61^

In Sweden for example, non-EU/EEA IMGs have described the national licensing process in that country as being disorganized, bureaucratic, and stricter than the process undertaken by EU/EEA IMGs.^61^ Others have suggested that the Swedish experience is not unique in Europe.^62^ In relation to this form of NLE approach, and unlike the previous approaches described, it is important to note that there is a lack of readily available research from which conclusions can be drawn.

Finally, jurisdictions such as Kuwait and Malta have eschewed NLEs as a means of regulating entry into the profession for both those prospective doctors who have trained in that jurisdiction and for IMGs.

## Discussion

It is clear that the landscape of NLEs internationally is varied. The literature reveals four different approaches. These range from all prospective doctors regardless of where they qualify in the world having to take the same examinations through to an absence of national licensing entirely.

Conceptualizing national licensing in terms of a candidacy typology allows us to explore the implications for medical standardization in the context of the internationalization of medical education. The standard economic model for understanding the impact of occupational regulation rests on the notion that there is a trade-off between the cost of service provision and its quality, ie, licensing increases the costs but also the quality of service. Shapiro developed this model, arguing that although regulating professions does increase the overall costs of service provision, it decreases the marginal costs of providing a quality service as it encourages investment in human capital (ie, better and more efficient training).^63^ However, the internationalization of medicine makes the direct application of this model more difficult, as licensing may result in a net shortage of qualified doctors, reducing the quality of service no matter how well trained the available pool of doctors.^64^

Those countries with across the board licensing, our second category, clearly have the problem of sustaining the recruitment of foreign doctors required to meet the demands of healthcare provision; as such they apply a degree of pragmatism. The demands that arise from the international shortage of physicians^65^ mean IMGs are not always prevented from practicing prior to passing the NLE in their chosen jurisdiction. Within North America there is an extensive support system which assists IMGs in preparing for the NLEs that they must eventually pass.^66,67^ In Canada the national licensing system is, to a degree, circumvented by IMGs using provincial licensing to practice until they are able to get full Licentiate of the Medical Council of Canada. The various forms of provisional licensing are used to balance the need for standardization against the need to cover doctor shortfalls within rural provinces.^66^ Thus, from a workforce planning perspective, introducing national licensing may be a more onerous process than developing the licensing assessments themselves. IMG doctors are likely to be disadvantaged, regardless of medical competence, as they are less familiar with the language and educational system.^68^

The third category of our typology, where IMGs have to sit some sort of licensing exam when their qualifications are deemed incompatible with national regulations, face similar issues, but these systems are generally more versatile and amenable to adaptation and change. In Australia, IMGs are offered relatively easy access to temporary licenses within specialties in which they have particular needs, or like Canada, in rural areas facing acute doctor shortages. Unlike Canada, however, there is less of an assumption that the IMGs will become permanent citizens.^69^

In the United Kingdom, Tiffin and colleagues note that even when other factors are accounted for, IMG doctors who have taken the Professional and Linguistic Assessments Board (PLAB) exam perform less well than their UK trained counterparts in their subsequent annual review of competence progression.^70^ This supports the findings of McManus and Wakeford who found that IMGs who had taken the PLAB test had a lower median score on subsequent specialty examinations than UK educated students.^71^ One way of providing standardization would be to significantly raise the bar of the PLAB test, as the GMC have sought to do with their review of the PLAB.^72^ However, as Tiffin et al note, this runs the risk of, “severe workforce planning challenges for the NHS, which has traditionally relied on IMGs” (p. 7).^70^ Similarly, in their analysis of the recruitment and licensing of physicians in Israel, Kugler and Sauer note that the problem of physician shortage points to an optimum level of licensing, whereby “stricter licensing requirements may lead to … lower average quality of service” (p. 437).^64^

The broader point here is that, in highly developed countries, national licensing systems have to operate within the context of doctor shortages and the increasing mobility of the global medical workforce. The candidacy typology developed here helps us to understand the international landscape in light of these dynamics, and draw out the implications of national licensing systems for workforce planning under different systems of licensure.

Summarizing NLEs internationally does have the risk of ignoring the cultural and historical subtleties of different nations. Some nation states have sought solutions to the enormity of their jurisdiction such as in North America where the State and Federal systems have been mirrored in their approach to medical regulation. In Europe, the principle of free movement includes doctors, but at the same time nation states increasingly are interested in protecting their own publics.

This study is also limited in that we were only able to gain access to information on 23 of the 49 countries comparable in terms of human development as ranked by the United Nations Development Programme (UNDP). However, given that the 23 countries on which we were able to gather data included the largest healthcare systems in the world’s biggest economies, it was a sufficient number to develop a typology and we are confident that other systems in highly development countries would fall into one of our four categories we have presented here.

Another limitation of this study is that it views these issues solely from the perspective of highly developed countries. Access to a sufficient medically-trained workforce is one of the factors that can be used to distinguish developed from developing countries.^73^ Thus the increasing mobility of the medical workforce has important implications for the developing world, in particular the “brain drain” that occurs when doctors travel to work oversees,^74,75^ which has been described as an “obstacle to global health.”^74^ The perspective of the developing world in terms of the impact of national licensing and other systems that affect the flow of doctors across borders is of course equally important.

## Conclusion

The drive towards standardization in medical regulation can be understood as a response to the internationalization of medical training and, more generally, the increasing mobility of the medical workforce in a globalized world. The typology developed here facilitates a comparison across systems and draws out the implications of these systems for workforce planning. Ultimately this gives an insight into the challenges that will shape the future debates around national licensing as a means of regulating medicine in a globalized world.

While national licensing may go some way towards reassuring publics of minimum standards in medical regulation, policy-makers and regulators in highly developed countries will need to consider the relative merits of national licensing options in relation to the need to address workforce planning issues where doctor shortages in that country necessitate flows of workers from overseas. Future research should therefore not only be directed at the effectiveness of national licensing to drive up standards, but integrated with broader questions concerning workforce needs and the compatibility of national licensing with workforce planning policy.

## Ethical issues

Not applicable.

## Competing interests

This study was funded by a tendered grant from the General Medical Council. The funders had no role in study design, data collection, and analysis, the decision to publish or preparation of the manuscript. Prof. Julian Archer (GMC No. 4438050) and Dr. Tom Gale (GMC No. 4095851) are licensed medical practitioners with the General Medical Council.

This is an academic paper based on material gleaned from a large systematic review for the GMC. The report is published online by the GMC at: http://www.gmc-uk.org/A_systematic_review_on_the_impact_of_licensing_examinations__for_doctors_in_countries_comparable_to_the_UK.pdf_61103496.pdf. While this paper arises from the report, it is qualitatively different and complementary.

## Authors’ contributions

JA and SRdB led the response to tender for the General Medical Council for funding. All members of the team developed and refined the search strategy. NL undertook the literature review with double reviewers and expert input from JA, MR, and LC. TP wrote the first draft of the paper, with substantial input from JA, with all authors contributing to the analysis and interpretation of the data from the review and the drafting of the final text.

## Authors’ affiliations

^1^Collaboration for the Advancement of Medical Education Research & Assessment (CAMERA), University of Plymouth, Plymouth, UK. ^2^Peninsula Schools of Medicine & Dentistry, University of Plymouth, Plymouth, UK. ^3^School of Medicine, Cardiff University, Wales, UK.

## Supplementary files

Supplementary file 1. Data extraction form.

Supplementary file 2. Letter to medical regulators.

Supplementary file 3. Survey of medical regulators.

Supplementary file 4. Records included and reviewed.
