# Supplementary material for: The International Landscape of Medical Licensing Examinations: A Typology Derived From a Systematic Review
Source: Int J Health Policy Manag. 2018 Apr 28;7(9):782–90. doi: 10.15171/ijhpm.2018.32 (PMC6186476; doi:10.15171/ijhpm.2018.32)
Supplement: Supplementary file 1 — Data extraction form. [file ijhpm-7-782-s001.pdf]

Supplementary file 1. Data Extraction Form

Paper number	
Date of extraction	
Reviewer	
Bibliographic details of study	
Purpose of study	
Methodological or Statistical techniques used	
**P**opulation	
**I**ntervention & Setting	
**C**omparison/Main alternative being considered	
**O**utcomes/Conclusions	
**S**tudy design/characteristics	
Evidence for **Content**	
Evidence for **Response Process**	
Evidence for **Internal structure**	
Evidence for **Relationship to other variables**	
Evidence for **Consequences**	
