# Supplementary material for: The International Landscape of Medical Licensing Examinations: A Typology Derived From a Systematic Review
Source: Int J Health Policy Manag. 2018 Apr 28;7(9):782–90. doi: 10.15171/ijhpm.2018.32 (PMC6186476; doi:10.15171/ijhpm.2018.32)
Supplement: Supplementary file 2 — Letter to medical regulators. [file ijhpm-7-782-s002.pdf]

Supplementary File 2. Letter to Medical Regulators

Dear ,

The Collaboration for the Advancement of Medical Education Research & Assessment (CAMERA)* have been commissioned by the General Medical Council (GMC) to carry out a review of the evidence for the impact of licensing examinations for doctors around the world. As part of the research we are conducting a survey and we are contacting you because you are a medical regulator or licensing examination provider. We are aware that you may not have a national licensing examination in your country but we would still like to hear from you.

The purpose of the survey is to learn about why you have or do not have a national licensing examination, your thinking and reasoning towards national licensing and to locate empirical evidence and informed opinion from all sides of the licensing debate. We are keen to know what evidence or research your approach to national licensing is based upon.

We would be very grateful for your input and so kindly invite you to complete the short attached survey and return it to camera.pupsmd@plymouth.ac.uk by give date.

Should you have any queries about the research including the survey, please do contact Dr Julian Archer at CAMERA or Name at GMC. Thank you in advance.

Yours sincerely,

Dr Julian Archer MRCPCH, MEd, PhD, FAcadMEd
NIHR Career Development Fellow,
Clinical Senior Lecturer & Director of the Collaboration for the Advancement of Medical Education Research & Assessment (CAMERA),
Plymouth University Peninsula Schools of Medicine & Dentistry,
C521 Portland Square,
Plymouth University,
Drake Circus,

Plymouth,
PL4 8AA
Tel No + 44 (0)1752 586750
Fax No + 44 (0)1752 586788
julian.archer@plymouth.ac.uk

*The Collaboration for the Advancement of Medical Education Research & Assessment (CAMERA), Plymouth University Peninsula Schools of Medicine and Dentistry, UK is a leading educational research group specialising in healthcare assessment and education including regulation, selection and remediation. We have previously completed research for the NIHR, GMC, the Medical Council of Ireland, the Department of Health in the UK, and the UK Health Foundation.
