# Supplementary material for: The International Landscape of Medical Licensing Examinations: A Typology Derived From a Systematic Review
Source: Int J Health Policy Manag. 2018 Apr 28;7(9):782–90. doi: 10.15171/ijhpm.2018.32 (PMC6186476; doi:10.15171/ijhpm.2018.32)
Supplement: Supplementary file 3 — Survey of medical regulators. [file ijhpm-7-782-s003.pdf]

Supplementary File 3. Survey of Medical Regulators

Survey to explore the validity of jurisdiction-wide licensing examinations

We are contacting you as a medical regulator or because you deliver large scale licensing examinations.

The General Medical Council (UK) has agreed to support the principle of establishing a UK national licensing examination. We have commissioned our research group at Plymouth University Peninsula Schools of Medicine and Dentistry to help develop the evidence base for large-scale medical licensing examinations. We are undertaking a systematic review of the published and grey literature and are contacting you to ask if you would be kind enough to complete this survey and help us identify the available evidence.

We are interested in the validity evidence for jurisdiction-wide examinations that doctors must successfully undertake in order to work in that jurisdiction. This includes any examinations directed at those who graduated in other jurisdictions (international medical graduates – IMGs). We are conscious that some of you might not have such examinations. We still want to hear from you however, as we are keen to understand your perceptions and experiences on this subject.

Please would you be kind enough to complete the questions below. We recognise that you might want to involve colleagues in completing some or all of the questions.

Your responses will be treated in the strictest confidence by the research team and the GMC.

Please return the survey to camera.pupsmd@plymouth.ac.uk by

Thank you in advance

Dr Julian Archer, Director of CAMERA (Collaboration for the Advancement of Medical Education Research & Assessment), NIHR Fellow and Clinical Senior Lecturer, Plymouth University Peninsula Schools of Medicine and Dentistry.

Your answers to the following questions might be supported by papers and reports that you hold either on or offline. We would be grateful if you were to add suitable web links to your answers or send us any offline material.

---

1. Do you have or are you in the process of introducing a jurisdiction-wide licensing examination for doctors? By this we mean an examination that must be passed by candidates in order to practise substantively (for example not on a short term fellowship) as a doctor in your jurisdiction. □ YES □ NO

If NO please answer questions 2 to 7 and 29 ONLY. If YES, please answer questions 8 onwards.

---

2. Please explain briefly the reasons why you do not have a jurisdiction-wide licensing examination.

3. Have you considered/tried implementing one in the past? □ YES □ NO
   If yes, please explain why it has not been implemented

4. What are the arguments in favour of a jurisdiction-wide licensing examination in your setting?

5. What are the arguments against a jurisdiction-wide licensing examination in your setting?

6. As you do not have a jurisdiction-wide licensing examination, how do you determine if a doctor with qualifications from *inside* your jurisdiction should be licensed and allowed to practise?

7. As you do not have a jurisdiction-wide licensing examination, how do you determine if a doctor with qualifications from *outside* your jurisdiction should be licensed and allowed to practise?

You have a jurisdiction-wide medical licensing examination

8. Please explain briefly why you have a jurisdiction-wide licensing examination

9. What do you see as the advantages of having a jurisdiction-wide licensing examination?

10. Do you think there are any weaknesses/disadvantages?

11. What does the assessment cover in terms of knowledge, clinical skills, professionalism, values etc.?

12. Is the format of the examination the same for home and international candidates? ◻ YES ◻ NO **Please give details**

13. How does the content of your examination relate to your medical school curricula and assessments, if at all?

14. At what point in medical training is the examination normally taken?

15. How do you ensure that the examination is set at the appropriate level?

16. What standard setting approaches do you use?

17. What measures are used to assess the reliability of the examination and what levels of reliability are typically achieved (for example Cronbach's alpha of 0.8)?

18. What are your annual pass and fail rates?

19. Are there opportunities to re-take the examination if failed on the first attempt?

20. How many home and how many international graduates take the examination each year?

21. How is it funded?

22. How much does it actually cost to run per candidate (if the figure is readily available)?

23. How is the examination regulated?

24. Do you have any evidence that performance in the examination:
    a. Is correlated with performance in medical school examinations?

b. Can it predict candidates' subsequent performance in clinical training and practise?

c. Can it predict the likelihood of referral to disciplinary proceedings for poor conduct or impaired practise?

d. Drives the ranking of organisations and individuals?

If you answer YES to any of the above please identify the source of that evidence

25. Do you have any evidence that your examination has:
    a. Led to better skilled registrants?

    b. Led to higher standards of practise?

    c. Reduced variation in undergraduate curricula between medical schools?

    d. Affected the supply of home or international medical graduates?

If you answer YES to any of the above please identify the source of that evidence.

26. Is the examination used as a quality assurance mechanism for medical school education?

27. Are candidates' scores ranked to support recruitment into further training or employment?

28. Do you have evidence of any differential pass rates or exam performance for candidates with different characteristics (such as home vs. international medical graduates or differential performance relating to gender, ethnicity, disability or age)?

29. Any other comments?

Thank you for completing the survey

Finally:

Please fill in the names and email contact details of those who completed the form. We may contact you to clarify any points.

Would you like the GMC to send you a link to the research report when completed?

☐ YES ☐ NO

Please return the survey to camera.pupsmd@plymouth.ac.uk by
