# Supplementary material for: The International Landscape of Medical Licensing Examinations: A Typology Derived From a Systematic Review
Source: Int J Health Policy Manag. 2018 Apr 28;7(9):782–90. doi: 10.15171/ijhpm.2018.32 (PMC6186476; doi:10.15171/ijhpm.2018.32)
Supplement: Supplementary file 4 — Records included and reviewed. [file ijhpm-7-782-s004.pdf]

Supplementary File 4. Records Included and Reviewed

1. Ahn DS, Ahn S. Reconsidering the Cut Score of the Korean National Medical Licensing Examination. J Educ Eval Health Prof. 2007;4(1):1-6. doi:10.3352/jeehp.2007.4.1

2. Committee to Evaluate the USMLE Program. Comprehensive Review of USMLE Summary of the Final Report and Recommendations. 2008.

3. Cuddy MM, Swanson DB, Dillon GF, Holtman MC, Clauser BE. A multilevel analysis of the relationships between selected examinee characteristics and United States Medical Licensing Examination Step 2 Clinical Knowledge performance: revisiting old findings and asking new questions. Acad Med. 2006;81(10 Suppl):S103-107.

4. Guttormsen S, Beyeler C, Bonvin R, et al. The new licencing examination for human medicine: from concept to implementation. Swiss Med Wkly. 2013;143:w13897. doi:10.4414/smw.2013.13897

5. Harik P, Clauser BE, Grabovsky I, Margolis MJ, Dillon GF, Boulet JR. Relationships among subcomponents of the USMLE Step 2 Clinical Skills Examination, the Step 1, and the Step 2 Clinical Knowledge Examinations. Acad Med. 2006;81(10 Suppl):S21-24. doi:10.1097/01.ACM.0000236513.54577.b5

6. Hecker K, Violato C. How much do differences in medical schools influence student performance? A longitudinal study employing hierarchical linear modeling. Teach Learn Med. 2008;20(2):104-113. doi:10.1080/10401330801991915

7. Holtzman KZ, Swanson DB, Ouyang W, Dillon GF, Boulet JR. International variation in performance by clinical discipline and task on the United States medical licensing examination step 2 clinical knowledge component. Acad Med. 2014;89(11):1558-1562. doi:10.1097/acm.0000000000000488

8. Green M, Jones P, Thomas JX, Jr. Selection criteria for residency: results of a national program directors survey. Acad Med. 2009;84(3):362-367. doi:10.1097/ACM.0b013e3181970c6b

9. Kenny S, McInnes M, Singh V. Associations between residency selection strategies and doctor performance: a meta-analysis. Med Educ. 2013;47(8):790-800. doi:10.1111/medu.12234

10. Kugler AD, Sauer RM. Doctors without Borders? Relicensing Requirements and Negative Selection in the Market for Physicians. J Labor Econ. 2005;23(3):437-465. doi:10.1086/430283

11. Lillis S, Stuart M, Stuart N. New Zealand Registration Examination (NZREX Clinical): 6 years of experience as an Objective Structured Clinical Examination (OSCE). N Z Med J. 2012;125(1361):74-80.

12. Margolis MJ, Clauser BE, Winward M, Dillon GF. Validity evidence for USMLE examination cut scores: results of a large-scale survey. Acad Med. 2010;85(10 Suppl):S93-97. doi:10.1097/ACM.0b013e3181ed4028

13. McManus IC, Wakeford R. PLAB and UK graduates' performance on MRCP(UK) and MRCGP examinations: data linkage study. BMJ. 2014;348:1-24. doi:10.1136/bmj.g2621

14. Musoke. 'Foreign Doctors and the Road to a Swedish Medical License.' 2012.

15. Norcini JJ, Boulet JR, Opalek A, Dauphinee WD. The relationship between licensing examination performance and the outcomes of care by international medical school graduates. Acad Med. 2014;89(8):1157-1162. doi:10.1097/acm.0000000000000310

16. Ranney RR. What the available evidence on clinical licensure exams shows. J Evid Based Dent Pract. 2006;6(1):148-154. doi:10.1016/j.jebdp.2005.12.012

17. Seyfarth M, Reincke M, Seyfarth J, Ring J, Fischer MR. Grades on the second medical licensing examination in Germany before and after the Licensing Reform of 2002: a study in two medical schools in Bavaria. Dtsch Arztebl Int. 2010;107(28-29):500-504. doi:10.3238/arztebl.2010.0500

18. Kenny S, McInnes M, Singh V. Associations between residency selection strategies and doctor performance: a meta-analysis. Med Educ. 2013;47(8):790-800. doi:10.1111/medu.12234

19. Stewart CM, Bates RE Jr, Smith GE. Relationship between performance in dental school and performance on a dental licensure examination: an eight-year study. J Dent Educ. 2005;69(8):864-869.

20. Sutherland K, Leatherman S. Regulation and Quality Improvement a review of the evidence. 2006.

21. Tamblyn R, Abrahamowicz M, Dauphinee D, et al. Physician scores on a national clinical skills examination as predictors of complaints to medical regulatory authorities. Jama. 2007;298(9):993-1001. doi:10.1001/jama.298.9.993

22. Tiffin PA, Illing J, Kasim AS, McLachlan JC. Annual Review of Competence Progression (ARCP) performance of doctors who passed Professional and Linguistic Assessments Board (PLAB) tests compared with UK medical graduates: national data linkage study. BMJ. 2014;348. doi:10.1136/bmj.g2622

23. Wenghofer E, Klass D, Abrahamowicz M, et al. Doctor scores on national qualifying examinations predict quality of care in future practice. Med Educ. 2009;43(12):1166-1173. doi:10.1111/j.1365-2923.2009.03534.x

24. Zahn CM, Saguil A, Artino AR Jr, et al. Correlation of National Board of Medical Examiners scores with United States Medical Licensing Examination Step 1 And Step 2 scores. Acad Med. 2012;87(10):1348-1354. doi:10.1097/ACM.0b013e31826a13bd
